# Toward Free‐Breathing Renal CEST MRI: Preclinical Evaluation of a Novel Prospective Respiratory Triggering Approach

**DOI:** 10.1002/mrm.70435

**Published:** 2026-05-17

**Authors:** Patrik Jan Gallinnis, Martin Duša, Julia Stabinska, Pavla Volešák Francová, Ruslan Garipov, Margarita Tkachenko, Karl Ludger Radke, Gerald Antoch, Alexandra Ljimani, Hans‐Jörg Wittsack, Luděk Šefc, Anja Müller‐Lutz, Vít Herynek

**Affiliations:** ^1^ Center for Advanced Preclinical Imaging (CAPI) First Faculty of Medicine, Charles University Prague Czech Republic; ^2^ Department of Diagnostic and Interventional Radiology Medical Faculty and University Hospital Düsseldorf, Heinrich‐Heine‐University Düsseldorf Düsseldorf Germany; ^3^ F.M. Kirby Research Center for Functional Brain Imaging Kennedy Krieger Institute Baltimore Maryland USA; ^4^ Division of Nephrology, Department of Medicine The Johns Hopkins University School of Medicine Baltimore Maryland USA; ^5^ MR Solutions Ltd Surrey UK; ^6^ CARID Cardiovascular Research Institute Düsseldorf, University Hospital Düsseldorf, Heinrich‐Heine‐University Düsseldorf Germany; ^7^ Biomedical Physics Heinrich Heine University Düsseldorf Düsseldorf Germany

**Keywords:** abdominal CEST, chemical exchange saturation transfer, kidney, magnetization transfer imaging, respiratory triggering

## Abstract

**Purpose:**

Endogenous renal chemical exchange saturation transfer (CEST) imaging enables contrast‐agent‐free assessment of renal metabolism but is highly sensitive to respiratory motion. Existing approaches, such as timed breathing, require high patient compliance or preclinical mechanical ventilation, limiting clinical translation. We evaluated a free‐breathing prospective respiratory triggering method that allows continuous saturation during active breathing while adaptively aligning image acquisition with the expiratory phase.

**Methods:**

Prospective triggering was based on a modified real‐time respiratory signal, with a temporal shift continuously updated according to the current respiratory rate. Simulations assessed its performance across saturation durations of 1–6 s, respiratory rates representative of preclinical and clinical settings (f = 8–90 bpm), and varying respiratory frequency (CV = 0%–25%). Results were compared to conventional real‐time triggering. For in vivo validation, prospective triggering was implemented on a preclinical 7 T MRI system using a Raspberry Pi‐based hardware and software. Six mice underwent CEST imaging with saturation durations of 1, 3, and 5 s using both real‐time and prospective triggering, and the Mean Squared Error of Lorentzian fit was used to compare the two approaches.

**Results:**

The real‐time trigger error shows a periodic dependence on respiratory frequency, which diminishes with increasing variability, whereas the prospective trigger reduces this periodicity. In vivo, prospective triggering significantly (*p* < 0.05) reduced motion‐induced scattering in the Z‐spectrum for saturation times up to 3 s and enabled the detection of distinct metabolic contrasts across renal compartments.

**Conclusion:**

Prospective triggering effectively reduces motion artifacts in preclinical renal CEST imaging.

## 1 Introduction

Endogenous renal chemical exchange saturation transfer (CEST) imaging is a promising MRI‐based technique for assessment of renal metabolic processes [1]. CEST imaging relies on the acquisition of a series of images with varying saturation offsets. Based on the signal intensity of a certain pixel in this image series, a so‐called Z‐spectrum is generated, from which metabolic information can be derived. The examination requires the kidney to remain spatially stable throughout the image acquisition. Otherwise, signal intensities from different tissue locations are combined, leading to data scattering [2, 3] and metabolic misinterpretation [4].

Respiratory motion thus represents a challenge in abdominal CEST imaging and has been addressed by timed‐breathing protocols in various studies, in which subjects synchronize their breathing with the image acquisition [3, 5, 6, 7]. This approach has allowed us to distinguish between healthy and pathological renal tissue [6] and the investigation of metabolic differences in native kidneys [3]. These studies demonstrate the feasibility of abdominal CEST imaging and also suggest that saturation can be applied throughout the active respiratory cycle, while the image needs to be acquired during the expiratory phase. This observation provides a conceptual basis for the development of novel respiratory motion correction strategies, which are sought in abdominal CEST studies [3, 5, 8, 9, 10].

Preclinical studies further highlight the potential of renal CEST imaging for assessing acute kidney injury (AKI) [11, 12, 13], ischemia [14], chronic kidney disease [15], diabetic kidney disease [10], acute renal allograft rejection [16] and pH‐mapping [17, 18, 19, 20]. However, translating these findings into clinical practice remains challenging, as preclinical studies typically rely on mechanically ventilated animals [11, 17], an approach that is difficult to justify in clinics. Clinical timed‐breathing protocols require high patient compliance and may be unsuitable for individuals prone to dyspnea.

**FIGURE 1:**
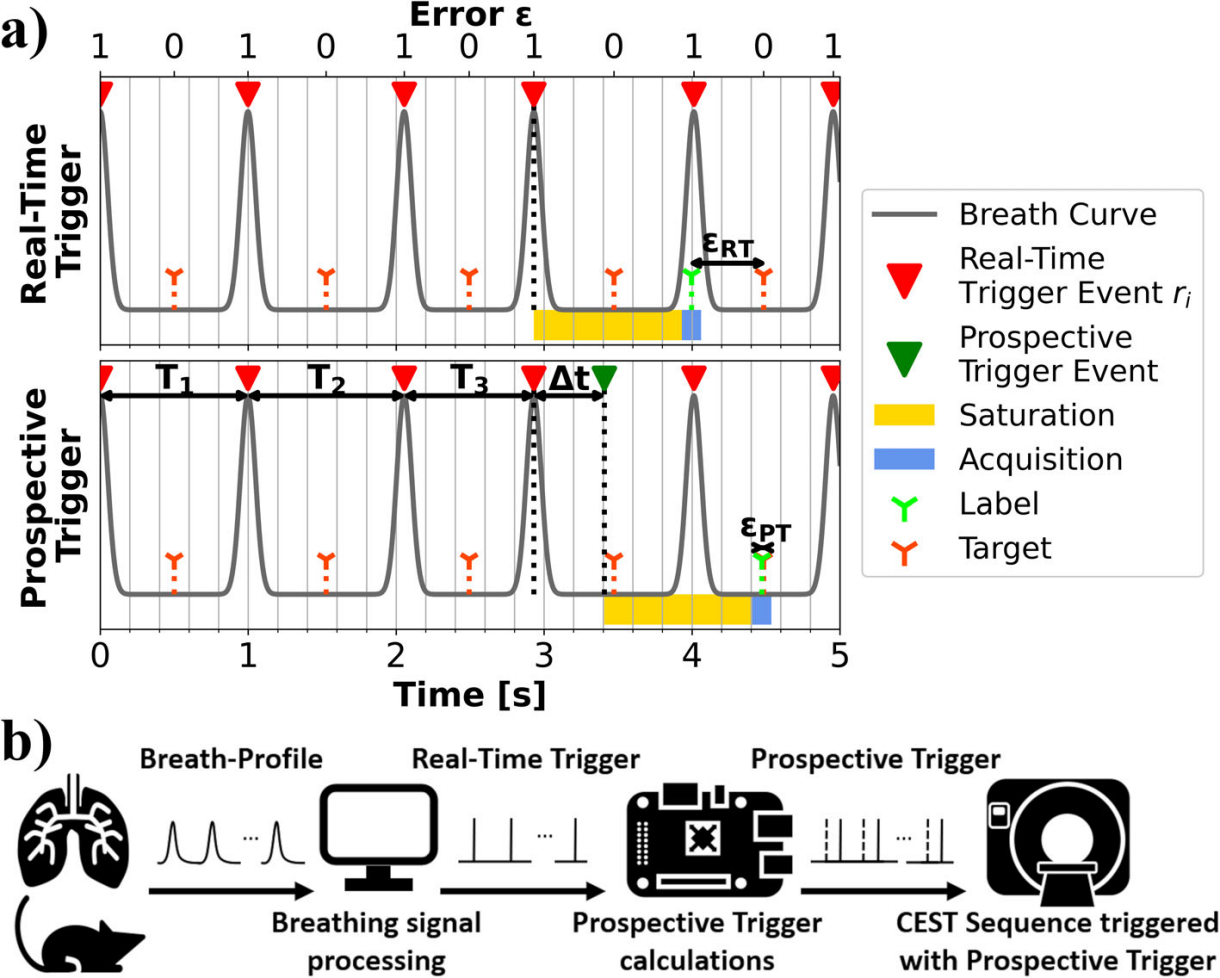
Based on the real‐time trigger (a, top), the temporal shift Δt is calculated from the first three respiratory periods (T_1_, T_2_, T_3_) of the real‐time trigger for prospective trigger (a, bottom). The temporal deviation between the center of the image acquisition (label) and the nearest center of the exhaled state (target) is then calculated for both the real‐time trigger and the prospective trigger. The error is scaled relative to the two real‐time trigger events from 0 to 1, where 0 indicates perfect acquisition in the exhaled state and 1 indicates maximal error (acquisition in the inhaled state). The example in (a) is a snapshot of the simulation with an average respiratory period T of 60 bpm, coefficient of variation CV of 12.5%, and a saturation time of 1 s, resulting in ε_RT_ of 0.96 and ε_PT_ of 0.03. For in vivo evaluation, a TTL‐based trigger signal was generated from the mouse respiratory profile to represent the real‐time trigger (b). For prospective triggering, this real‐time TTL signal was processed by a Raspberry Pi and transmitted to the scanner as the prospective trigger.

In both preclinical and clinical MRI, respiratory triggering is commonly used but is suboptimal for CEST imaging because the required saturation period often spans over multiple breathing cycles, which hinders reliable synchronization with expiration (Figure 1a). Although continuous saturation [8, 21], shortened saturation duration [9] or fixed temporal trigger shifts [10] can partially address this issue, these approaches either limit the saturation strategy or may fail for longer saturation durations and respiratory variability.

Therefore, we proposed a novel triggering approach based on a modified real‐time respiratory signal, in which the temporal shift is continuously updated to the current respiratory rate, allowing saturation throughout the active breathing cycle while prospectively aligning image acquisition with expiration (Figure 1). This prospective triggering was evaluated in simulations across different saturation durations, clinical and preclinical respiratory rates and variabilities, and compared to conventional real‐time triggering. Prospective trigger was implemented on a preclinical 7 T MRI system and validated in vivo on an animal model.

## 2 Methods

### 2.1 Prospective Trigger

The prospective trigger signal represents a temporally modified version of the real‐time respiratory trigger. Trigger events are shifted so that the center of the image acquisition coincides with the middle of the expiration phase, while accounting for both the saturation duration and acquisition time. The temporal shift applied to each trigger event is continuously updated based on the current respiratory period, estimated from the average of the three most recent real‐time trigger intervals, thereby increasing robustness to respiratory variability. The temporal offset Δt applied to each real‐time trigger event is calculated by

(1) $$\Delta t=\left(n\cdot \bar{T}-t_{\mathrm{sat}}\right)+\frac{\bar{T}-t_{\mathrm{acq}}}{2},\text{with}\;n=\left\lceil \frac{t_{\mathrm{sat}}}{\bar{T}}\right\rceil$$

where $\bar{T}$ is the respiratory period calculated as the mean of the three most recent trigger intervals, *t*_*sat*_ is the saturation duration, and *t*_*acq*_ is the acquisition time. The first addend shifts the acquisition prospectively to the beginning of the *n*‐th respiratory cycle, calculated by the ceiling operator ⌈·⌉, while the second addend centers the acquisition within the expiration phase (Figure 1a).

### 2.2 In Silico Simulation

To evaluate CEST acquisition timing and compare real‐time and prospective triggering under identical respiratory conditions, a simulation framework was implemented. Each simulated acquisition was based on a respiratory scenario defined by real‐time trigger events $\mathrm{RT}=\left(r_{i}\right)$, where each $r_{i}$ corresponds to maximal inhalation (Figure 1). The sequence was generated recursively as $r_{i}=r_{i-1}+T_{i}$, with breathing periods $T_{i}$ drawn from a lognormal distribution [22] using *numpy.random.lognormal*($\mu_{\ln},\sigma_{\ln}$) to model respiratory variability, with the expected mean value $\mu_{\ln}$ and standard deviation $\sigma_{\ln}$. The distribution parameters $\mu_{\ln}$ and $\sigma_{\ln}$ were derived from the mean respiratory period T and the coefficient of variation (CV) by $\sigma_{\ln}=\sqrt{\ln\left(1+{\mathrm{CV}}^{2}\right)}\;\text{and}\;\mu_{\ln}=\ln\left(T\right)-\frac{1}{2}\sigma_{\ln}^{2}$.

To calculate the prospective triggering acquisition timing, the temporal shift Δt was calculated using Equation (1) based on the saturation duration (t_sat_), acquisition time (t_acq_), and the respiratory period $\bar{T}$ estimated by averaging the first three breathing periods. In real‐time triggering, acquisition timing was calculated directly to the real‐time trigger event. Timing accuracy was quantified by the error ε, defined as the normalized difference between the acquisition center and the nearest exhaled state (Figure 1a). Simulations covered breathing rates from 8 bpm (resting humans) to 90 bpm (lightly anesthetized B6 mice [23]) with CV ranging [24] from 0 to 0.25.

Acquisition time was fixed at 131 ms, and the number of simulated frequency offsets was set to 73, consistent with the optimized in vivo CEST sequence, while saturation duration was varied between 1 and 6 s.

### 2.3 Hardware and Software Adaptions for Implementing Prospective Triggering

The TTL trigger signal from the breathing monitoring system was processed via the GPIO interface of a Raspberry Pi, where the temporally shifted trigger was computed and forwarded to the MRI scanner. To minimize latency, a multithreaded software architecture was used. One thread continuously acquired trigger events, estimated the respiratory period by averaging the last three trigger intervals, and computed the temporal offset *Δt* (Equation 1), while a second thread generated the delayed TTL output.

### 2.4 In Vivo Experiments

In vivo experiments were performed on a 7 T preclinical MRI system (MRS*Drymag 7017PW, MR Solutions, Guildford, UK) with a mouse body coil. For each scan, RF calibration with a stimulated‐echo method [25, 26] and manual B_0_ shimming were performed.

Morphological images were acquired using a fast spin‐echo sequence for CEST planning and segmentation (coronary FOV 20.0 × 10.0 × 1.5 mm [3], matrix size of 128 × 128, TE/TR = 6.0/5000 ms, turbofactor TF = 32, 3 averages, acquisition bandwidth 33.3 kHz). For CEST imaging, the matrix was reduced to 64 × 64 (TE/TR = 4.09/5500 ms, TF = 32, one average). Each CEST image acquisition was divided into two CEST‐sequence blocks, each independently triggered to distinct exhalation phases and acquiring half of k‐space within 131 ms. The TR was defined according to literature recommendations [19]. In the context of respiratory triggering, TR denotes the minimum interval between the start of consecutive CEST‐sequence blocks. Saturation was applied using a 1.5 μT block pulse with durations of 1, 3, and 5 s. Z‐spectra were acquired at 73 frequency offsets (±1800 Hz/˜6.0 ppm, 50 Hz/˜0.17 ppm steps), resulting in a protocol duration of 13 min and 23 s (˜14.5 min with real‐time or prospective triggering). A real‐time triggered image without saturation was acquired for normalization.

Six healthy adult female B6 albino mice were scanned using both real‐time and prospective triggering for *t*_*sat*_ = [1, 3, 5] s. For reproducibility assessment, six healthy female BALB/c mice were scanned twice within the same imaging session using prospective triggering, with identical shimming and positioning, with t_sat_ of 3 s. Anesthesia was induced with 3.0% and maintained with 1.75%–2.0% isoflurane (Isothesia, Covetrus, Portland, USA) in air, maintaining respiratory rates within 40–60 bpm. Body temperature was kept at 37°C. Respiration was monitored using a small‐animal gating system (ERT Control/Gating Module, Model 1030, SA Instruments Inc., New York, USA), generating a TTL trigger signal that was either directly applied for real‐time triggering or routed through a Raspberry Pi for prospective triggering (Figure 1b).

All animal experiments were approved by the Laboratory Animal Care and Use Committee of the First Faculty of Medicine, Charles University, and the Ministry of Education, Youth and Sports of the Czech Republic (MSMT‐16533/2024‐5). The approved protocol was in accordance with the Act of the Czech Parliament for the Protection of Animals Against Cruelty No. 246/1992 and the Directive 2010/63/EU of the European Parliament.

### 2.5 Postprocessing

Data analysis and visualization were performed using custom Python scripts. For CEST analysis, the renal inner medulla (IM), outer medulla (OM), and cortex (CO) were manually segmented using ITK‐SNAP [27] and the kidney was cropped across the image series for an Image Downsampling Expedited Adaptive Least‐squares (IDEAL) fitting framework [18, 28] with a 5‐pool Lorentzian model [29],

(2) $$\hat{Z}\left(\omega \right)=Z_{\text{base}}-\sum_{i}^{\text{Pool}}L_{i}\left(\omega \right),\text{with}\;L_{i}\left(\omega \right)=A_{i}\cdot \frac{\;\frac{\Gamma_{i}^{2}}{4}}{\;\frac{\Gamma_{i}^{2}}{4}+{\left(\omega -\Delta \omega \right)}^{2}}$$

where A is the amplitude, Γ is the full‐width at half‐maximum (FWHM), Δω is the frequency offset of the CEST‐pool.

The cropped image series were spatially downsampled by averaging kernel using patch sizes of [1 × 1], [2 × 2], [4 × 4], [8 × 8], [16 × 16], and [32 × 32] pixels. For each patch, B_0_ inhomogeneity was estimated from the minimum of a spline‐interpolated Z‐spectrum [30] (scipy.interpolate.UnivariateSpline() [31], smoothing factor s = 0.01, interpolation step size = 0.01 ppm) and used as a fixed parameter for fitting.

The fitting was performed by nonlinear least‐squares optimization using the Trust‐Region‐Reflective algorithm (scipy.optimize.curve_fit() [31]) with initial values and bounds for water (A = [0.02;0.9;1.0], Γ = [0.3;1.4;10.0]), amine (A = [0.0;0.01;0.2], Γ = [1.0;1.5;3.5], Δω= [1.0;2.2;2.5]), amide (A = [0.0;0.025;0.2], Γ = [0.4;0.5;3.0], Δω = [3.0;3.5;4.0]), nuclear Overhauser effect (NOE) (A = [0.0;0.02;0.4], Γ = [1.0;3.0;5.0], Δω = [−4.5;‐3.5;2.0]), magnetization transfer (MT) (A = [0.0;0.1;1.0], Γ = [10.0;25.0;100.0], Δω = [−4.0;‐2.0;‐2.0]), and baseline ($Z_{\text{base}}$ = [0.5; 1.0; 1.0]) at 7 T were adopted from Windschuh et al. [29] In the IDEAL method, amplitude and FWHM were constrained to ±10% and frequency offsets to ±5% of the initial fit results [28].

As no fat suppression was applied and lipid signals may influence the NOE effect [32] around −3.5 ppm, the signal observed at this offset is referred to as pseudo‐NOE (pNOE) [33] in this study.

### 2.6 Statistics

Previous studies [2, 3] demonstrated that motion artifacts increased data‐scattering in the Z‐spectrum. Therefore, the mean squared error (MSE) between measured $Z\left(\omega_{i}\right)$ and Lorentzian‐fitted $\hat{Z}\left(\omega \right)$ spectra with 73 frequency offsets for each pixel were assessed to quantify motion in CEST data:

(3) $$\mathrm{MSE}=\frac{1}{73}\sum_{i=1}^{73}{\left(Z\left(\omega_{i}\right)-\hat{Z}\left(\omega_{i}\right)\right)}^{2}$$

ROI‐averaged MSE values for real‐time and prospective trigger were compared using the Wilcoxon signed‐rank test for each saturation duration.

For each CEST pool, the fitted amplitudes were averaged within the respective ROIs. The normality of the pairwise differences in ROI‐averaged amplitudes was assessed using the Shapiro–Wilk test (α = 0.01). Paired *t*‐tests with Bonferroni correction were performed to determine significant metabolic differences between the IM, OM, and CO for prospective triggering. In reproducibility experiments, fitted amplitudes were averaged within the respective ROIs and compared between measurements using intraclass correlation coefficient ICC(3,1) [34] and categorized as poor (< 0.5), moderate (< 0.75), good (< 0.9), or excellent (> 0.9). The MSE of the initial feasibility measurement using real‐time triggering was compared with the MSE from the reproducibility measurements using a Mann–Whitney test. Significance levels were marked by (****) for *p* ≤ 0.0001, (***) for *p* ≤ 0.001, (**) for *p* ≤ 0.01, (*) for *p* ≤ 0.05; *p* > 0.05 was considered as nonsignificant. All statistical analyses were performed using the Python library SciPy [31, 35].

## 3 Results

### 3.1 In Silico Simulation

**FIGURE 2:**
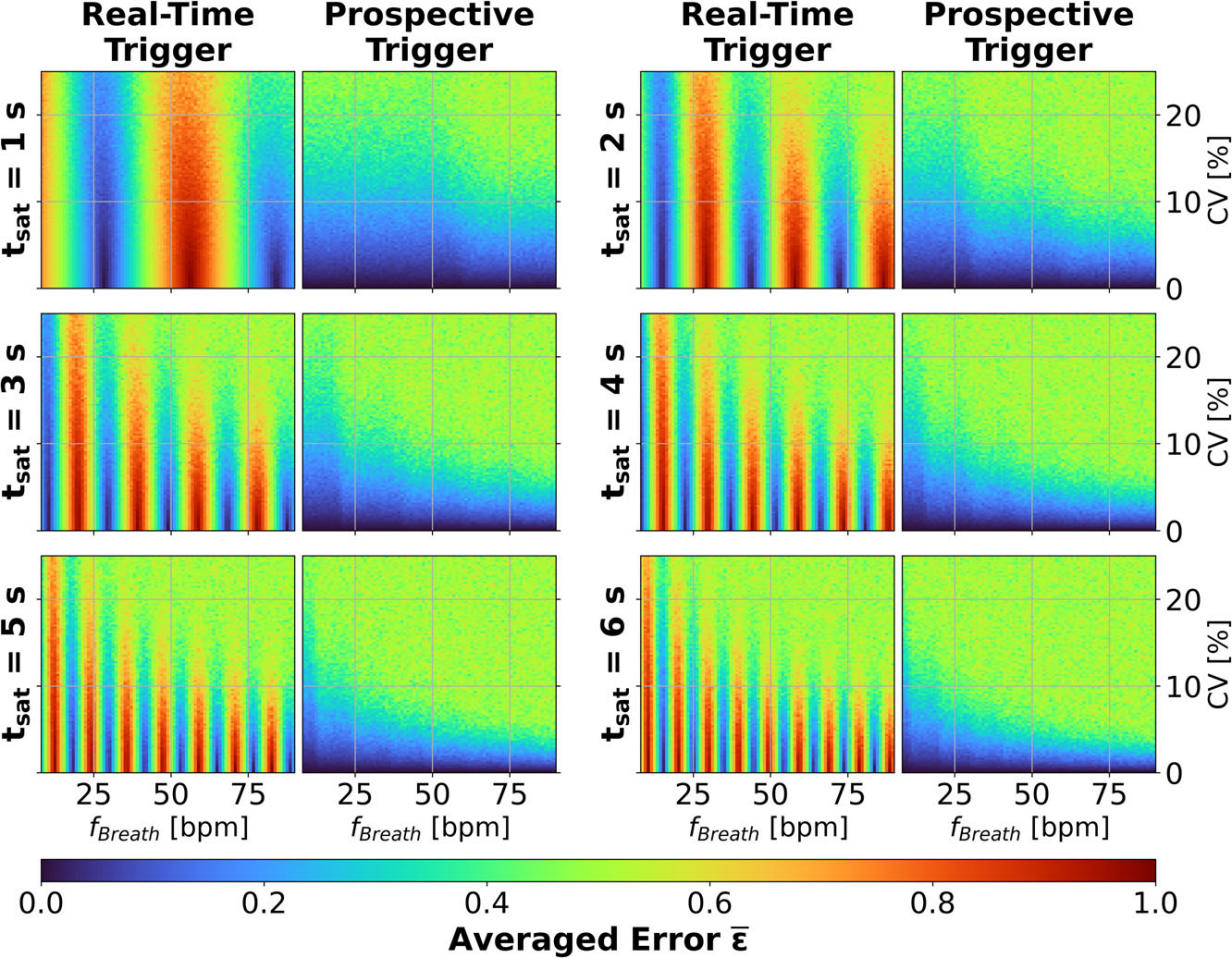
Simulated averaged error $\bar{\varepsilon }$ of the real‐time trigger and the prospective trigger in positioning image acquisition into the exhaled state. An error value of 1 indicates that image acquisition always occurs during the inhaled state, whereas an error value of 0 indicates that image acquisition always occurs during the exhaled state.

The error of the real‐time trigger exhibited a pronounced periodic dependence on respiratory frequency and reached its maximum at respiratory periods corresponding to multiples of the saturation time plus half of the acquisition time (Figure 2). With increasing variability of the respiratory frequency, the periodicity of the error decreased; this effect occurred earlier at higher respiratory frequencies than at lower frequencies. For the prospective trigger, the periodic dependence of the error was reduced, showing only minor stepwise increases in error at positions corresponding to the maxima observed for the real‐time trigger.

### 3.2 In Vivo Experiments

**FIGURE 3:**
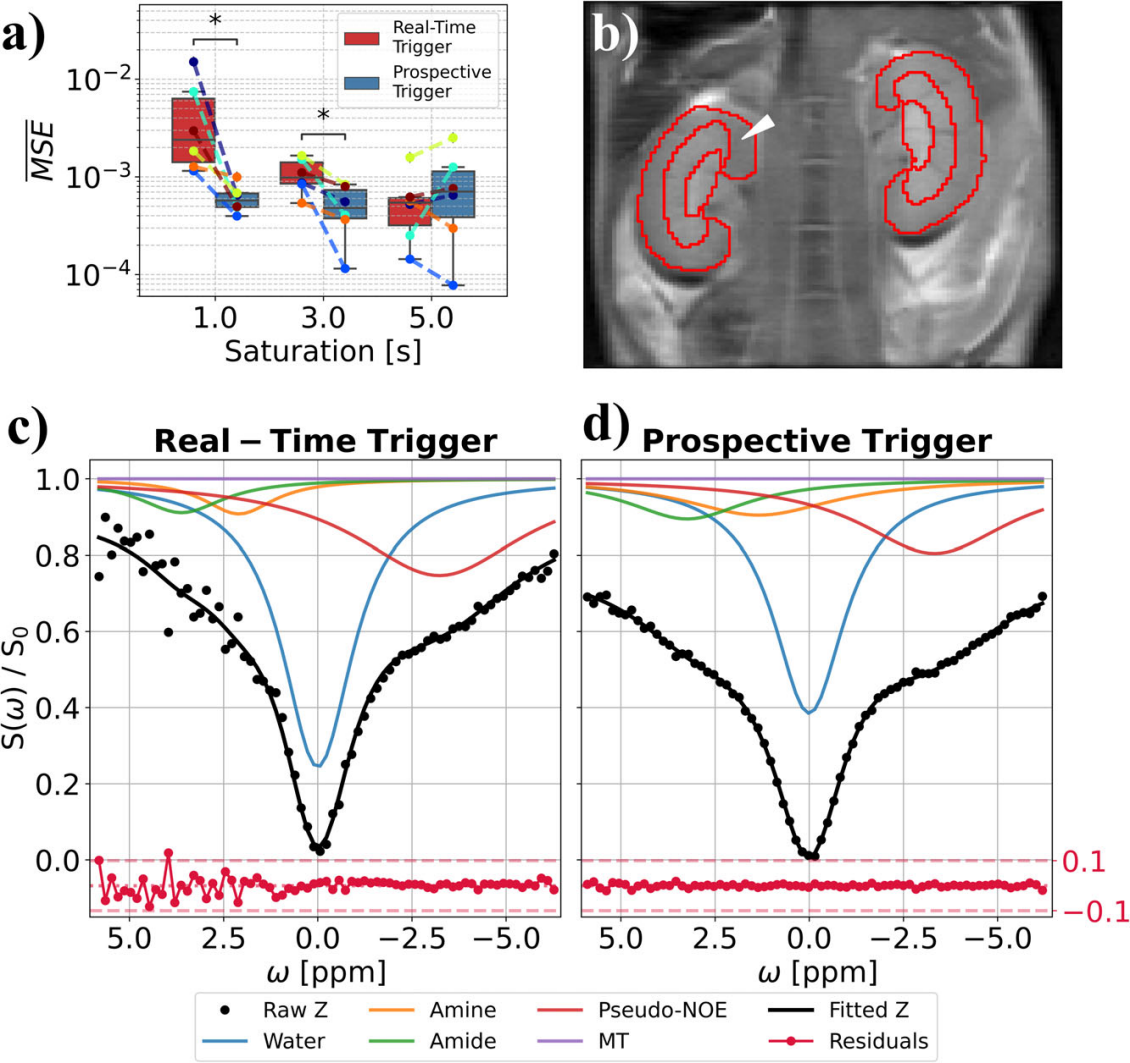
The mean MSE of the Lorentzian fit (a) for individual mice (each shown in a different color) was significantly (*p* < 0.05) reduced in the in vivo experiments for saturation times of 1 and 3 s, when using prospective triggering compared to real‐time triggering. In the segmentation process (b), a margin from the kidney boundary was maintained, particularly at borders perpendicular to the cranial‐caudal axis (frequency encoding direction), to minimize signal changes induced by water‐fat shift artifacts that could affect the IDEAL fitting results. A representative Z‐spectrum with a saturation time of 3 s from a single voxel in the segmented cortex exhibits pronounced data scatter and increased fitting residuals with real‐time triggering (c). In contrast, the Z‐spectrum from the same voxel acquired with prospective triggering shows a smoother spectral profile and reduced residuals (d).

Reduced motion was observed in the CEST image series with prospective triggering (Supporting Information, Video S1). Prospective triggering significantly (*p* < 0.05) reduced the MSE in the kidney (Figure 3a) compared to real‐time triggering for saturation durations of 1 s (from (4.96 ± 5.48) × 10^−3^ to (0.62 ± 0.21) × 10^−3^) and 3 s (from (1.09 ± 0.43) × 10^−3^ to (0.51 ± 0.28) × 10^−3^). No significant difference (*p* > 0.05) in MSE was found between prospective ((0.93 ± 0.88) × 10^−3^) and real‐time triggering ((0.61 ± 0.51) × 10^−3^) for 5 s saturation. An exemplary Z‐spectrum from a cortical pixel (Figure 3b) showed pronounced scattering between 6.0 and 1.0 ppm (Figure 3c), resulting in an increased MSE of 1.07 × 10^−3^. In contrast, the same pixel acquired with prospective triggering exhibited reduced scattering (Figure 3d) and a lower MSE of 0.05 × 10^−3^. Additionally, the Z‐spectrum obtained with prospective triggering displayed lower signal intensities at the spectral flanks and broader fitted pools compared to real‐time triggering.

**FIGURE 4:**
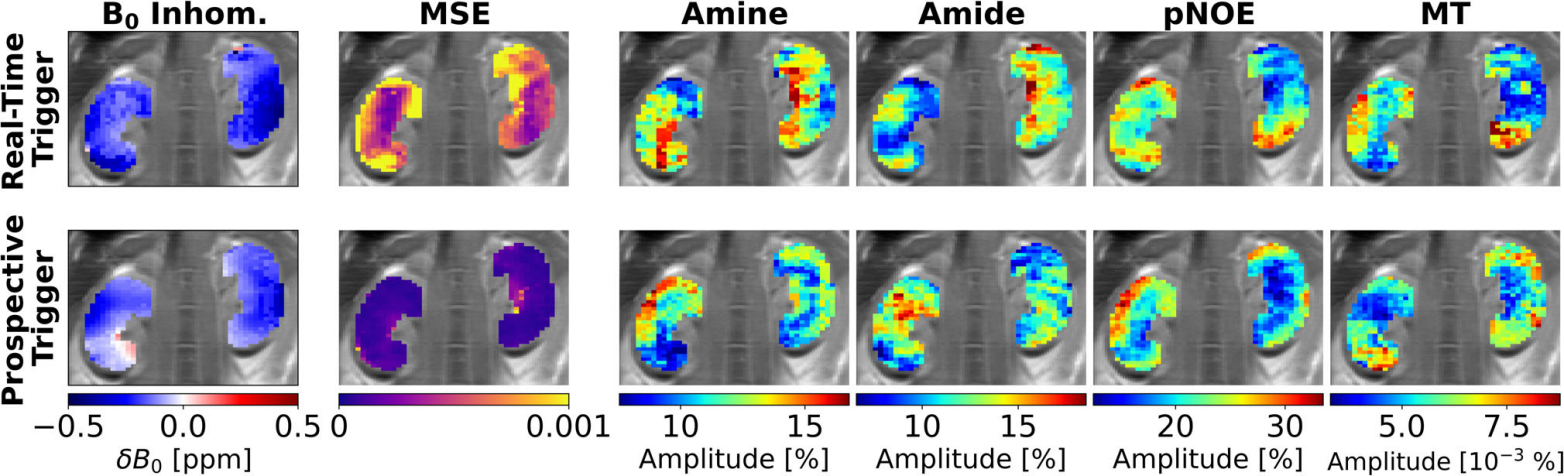
Exemplary B_0_ maps (left), Lorentzian fit MSE maps (second from left), and corresponding CEST contrast maps from in vivo measurements acquired with real‐time triggering (top row) and prospective triggering (bottom row) at a saturation duration of 3 s. For the exemplary maps, mean CEST amplitudes (mean ± SD) in inner medulla, outer medulla, and cortex were in corresponding order: amine: (8.79 ± 1.21) %, (7.81 ± 1.40) %, (8.91 ± 1.90) %; amide: (10.12 ± 1.33) %, (9.71 ± 1.11) %, (9.13 ± 1.20) %; pNOE: (15.36 ± 1.50) %, (16.28 ± 1.77) %, (18.94 ± 2.23) %; MT: (2.82 ± 0.24) × 10^−3^%, (3.13 ± 0.35) × 10^−3^%, (3.46 ± 0.36) × 10^−3^%.

B_0_ maps were generally homogeneous for both triggering approaches but appeared more spatially continuous with prospective triggering (Figure 4). The Lorentzian fit MSE decreased from (0.85 ± 2.71) × 10^−3^ with real‐time triggering to (0.12 ± 0.09) × 10^−3^ with prospective triggering. CEST contrast maps acquired prospectively showed clearer renal structure delineation, with medulla‐cortex differentiation visible in the amine, pNOE, and MT maps, whereas such features were less discernible in real‐time triggered data. Structural contrast was less pronounced in the amide maps. Localized cluster‐like variations were observed in the lower pole of the left kidney in amine maps and the upper pole of the right kidney in MT maps for prospective‐triggered images (Figure 4).

**FIGURE 5:**
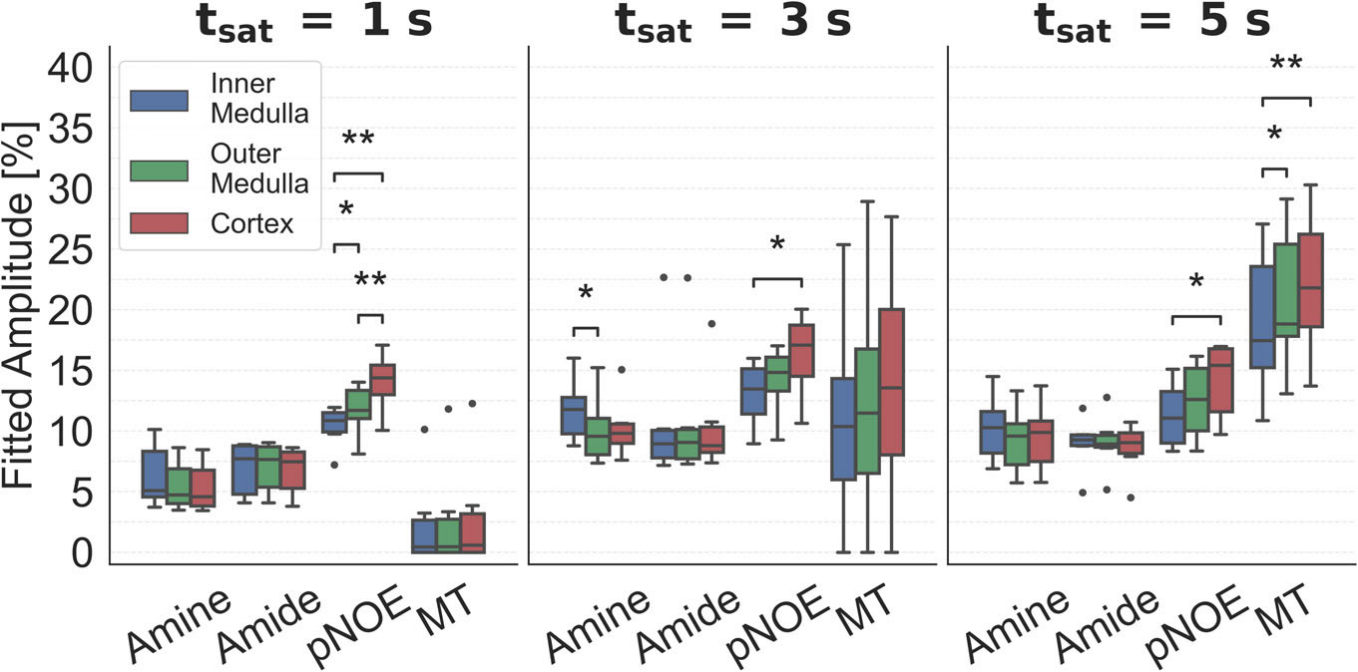
ROI‐averaged CEST effects from the in vivo experiments. Statistical significance was assessed using a paired *t*‐test with Bonferroni correction, with significance levels defined as (**) for *p* ≤ 0.01 and (*) for 0.01 < *p* ≤ 0.05. Box plot shows the median; dots represent outliers.

The Shapiro–Wilk test confirmed normal distribution of the data acquired with prospective triggering. Significant differences in CEST effects were observed between renal compartments (Figure 5). For all investigated saturation durations, the pNOE CEST effect differed significantly between IM and CO, with an additional significant difference between IM and OM at 1 s. At a saturation duration of 3 s, significant differences in the amine CEST effect were observed between IM and OM, whereas at 5 s, the MT pool showed significant differences between IM and both OM and CO.

MSE was significantly reduced (*p* < 0.05) compared to real‐time triggering, with MSE of (0.33 ± 0.17) × 10^−3^ and (0.49 ± 0.30) × 10^−3^ for the first and second reproducibility measurements, respectively. Reproducibility was poor for amide and NOE CEST effects (ICC(3,1) < 0.5) and for amine in the IM (ICC(3,1) = 0.47), moderate for MT effects (0.55 < ICC(3,1) < 0.66), and good for amine in the OM (ICC(3,1) = 0.76) and cortex (ICC(3,1) = 0.85).

## 4 Discussion

The prospective trigger was successfully evaluated in simulations, preclinical renal CEST imaging, and compared to conventional real‐time triggering.

In silico, the error of the prospective trigger increased stepwise at respiratory periods in which real‐time triggering performed worse. This behavior arises from the ceiling function in Equation (1), which shifts acquisition to the next respiratory cycle and thereby increases sensitivity to respiratory variability, as indicated by simulations at longer saturation times. Simulations show that prospective triggering reduces the influence of respiratory rate on successful image acquisition.

The quality of Z‐spectra was significantly improved using the prospective trigger in vivo for saturation times up to 3 s. For a saturation time of 5 s, no significant improvement was observed. This lack of significance is likely due to the variation in respiratory frequency over longer saturation times, which may cause the calculation of Δt based on the previous three breaths to fail in accurately predicting the duration of subsequent multiple breaths. In addition, the relative error between the two triggering approaches depends on the degree of synchrony between respiration and image acquisition, which may change during the measurement, as suggested by the Z‐spectra in Figure 3c, where motion‐induced data scatter is observed within a specific interval only. This effect has been investigated in vitro [3] and may contribute to the inter‐individual variability observed in the MSE. Saturation times over 4 s are mainly used in exogenous CEST imaging [12, 14, 16, 17], thus the applicability of prospective triggering may be limited in these protocols.

Increased CEST effects at 2.2 ppm in the IM and OM, potentially related to amine groups [36], are consistent with findings by Wang et al. [10] In contrast, amide signals showed no clear compartment‐specific pattern, in agreement with Zhang et al. [13] and Tao et al. [11], who investigated APT effects in AKI. Zhang et al. also showed a corticomedullary increase in rNOE toward the CO. However, the use of fat suppression in their study, in contrast to the present work, may explain the differences in the observed pNOE CEST effect [36].

Our study has limitations that are important to note. First, the CEST results depend on saturation parameters [37] and reflect a metabolic snapshot that may vary with hydration [38], anesthesia [39], circadian factors [40], and respiration‐dependent recovery times may influence the z‐spectra. In addition, arterial pulsation, peristaltic motion (Supporting Information, Video S1), and B_1_/B_0_ field alterations during respiration may contribute to the cluster‐like patterns observed in the CEST maps, likely arising from locally varying saturation efficiency and overly constrained fit boundaries in the IDEAL fitting process (e.g., amine map at the renal pole in Figure 2). Second, the reproducibility was limited, particularly for pNOE and amide, whereas MT showed predominantly moderate and amine largely good reproducibility. The observed differences may be attributed to temporal metabolic variations in the kidney, potentially related to anesthesia [39, 41, 42, 43, 44, 45], which hampers access to reproducibility measurements under consistent metabolic conditions. However, metabolic variability is unlikely to compromise the feasibility of prospective triggering, as MSE was reduced compared to real‐time acquisitions, including in reproducibility experiments. Future studies may further improve robustness by incorporating B_1_/B_0_ inhomogeneity correction methods [5, 46], saturation pulses less sensitive to field alterations [47], and assessing reproducibility in vitro using breathing phantoms under controlled metabolic conditions [3]. In vivo, residual abdominal motion may be mitigated by fasting or butylscopolamine [48] administration and further addressed using image‐based motion correction methods [49]. Third, the simulations represent a simplified model of respiratory triggering in CEST imaging, whereas clinical respiration may be more complex and irregular [24, 50], which has so far only been investigated in simulations.

Thus, future studies could validate prospective triggering in clinical in vivo settings. Implementation may be achieved through hardware‐based solutions, as demonstrated here, or software integration. Vendor‐independent frameworks such as Pulseq [37], which support external triggers, may further enable simulation‐driven optimization of triggering and saturation parameters for renal CEST imaging [37, 47, 51].

## 5 Conclusion

Prospective triggering effectively reduces motion artifacts in preclinical renal CEST imaging under free‐breathing conditions.

## Funding

This work was supported by Jürgen Manchot Stiftung, the Univerzita Karlova v Praze (SVV 260 635/2025), the Deutsche Forschungsgemeinschaft (530863408, NE 2136/3‐1), the Ministerstvo Školství, Mládeže a Tělovýchovy (LM2023050), and the European Regional Development Fund (CZ.02.1.01/0.0/0.0/18_046/0016045). Julia Stabinska was supported by the National Institutes of Health (K99 DK138294).

## Conflicts of Interest

Ruslan Garipov is an employee of MR Solutions.

## Supporting information


**Video S1:** This video compares a CEST image series acquired using prospective triggering and real‐time triggering.

**Data S1:** Program code.

## Data Availability

The Python code ProspectiveTrigger.py for computation of the trigger shift and generation of a prospective trigger signal is available in the Supporting Information. Other data are available upon request.

## References

[1] L. Liu , S. Guo , Z. Xing , et al., “Chemical Exchange Saturation Transfer Magnetic Resonance Imaging of the Kidney: Applications and Challenges,” Abdominal Radiology 50 (2025): 5934–5947, 10.1007/s00261-025-04980-2. 40448845 PMC12602669

[2] G. L. Simegn , A. J. W. Van der Kouwe , F. C. Robertson , E. M. Meintjes , and A. Alhamud , “Real‐Time Simultaneous Shim and Motion Measurement and Correction in glycoCEST MRI Using Double Volumetric Navigators (DvNavs),” Magnetic Resonance in Medicine 81, no. 4 (2019): 2600–2613, 10.1002/mrm.27597. 30506877 PMC7251754

[3] P. J. Gallinnis , B. Kamp , K. L. Radke , et al., “Investigation of Endogenous Renal CEST Contrast and the Influence of Respiratory Motion on a Clinical 3 Tesla MRI: An In Vivo and In Vitro Study,” Magnetic Resonance in Medicine 95, no. 4 (2026): 2194–2206, 10.1002/mrm.70210. 41340208 PMC12850607

[4] M. Zaiss , K. Herz , A. Deshmane , et al., “Possible Artifacts in Dynamic CEST MRI due to Motion and Field Alterations,” Journal of Magnetic Resonance 298 (2019): 16–22, 10.1016/j.jmr.2018.11.002. 30500568

[5] P. Bulanov , P. Menshchikov , J. A. Grimm , et al., “Human Liver CEST Imaging at 7 T: Impact of B1+ Shimming,” Magnetic Resonance in Medicine 94, no. 4 (2025): 1604–1615, 10.1002/mrm.30557. 40411372 PMC12309884

[6] X. Wang , J. Keupp , I. E. Dimitrov , et al., “Evaluation of Renal Masses With CEST: Protocol Optimization and Preliminary Results,” Magnetic Resonance in Medicine 94, no. 6 (2025): 2374–2387, 10.1002/mrm.30641. 40693363 PMC12501682

[7] X. Wang , Y. Y. Cao , Y. Jiang , et al., “Effects of Breathing Patterns on Amide Proton Transfer MRI in the Kidney: A Preliminary Comparative Study in Healthy Volunteers and Patients With Tumors,” Journal of Magnetic Resonance Imaging 60, no. 1 (2024): 222–230, 10.1002/jmri.29099. 37888865

[8] S. Mueller , R. Pohmann , R. Chiaffarelli , et al., “An Open Source Triggered CEST Module for Bruker Systems for Reliable CEST MRI With Efficient Motion Artifact Mitigation,” in Magnetic Resonance Materials in Physics, Biology and Medicine, vol. 34 (ESMRMB, 2021), S18–S19, 10.1007/s10334-021-00947-8.

[9] Z. Chen , C. Liu , Y. Wang , et al., “Free‐Breathing Abdominal Chemical Exchange Saturation Transfer Imaging Using Water Presaturation and Respiratory Gating at 3.0 T,” NMR in Biomedicine 37, no. 8 (2024): 2–3, 10.1002/nbm.5134. 38459747

[10] F. Wang , D. Kopylov , Z. Zu , et al., “Mapping Murine Diabetic Kidney Disease Using Chemical Exchange Saturation Transfer MRI,” Magnetic Resonance in Medicine 76, no. 5 (2016): 1531–1541, 10.1002/mrm.26045. 26608660 PMC4882276

[11] Q. Tao , Q. Zhang , Z. An , Z. Chen , and Y. Feng , “Multi‐Parametric MRI for Evaluating Variations in Renal Structure, Function, and Endogenous Metabolites in an Animal Model With Acute Kidney Injury Induced by Ischemia Reperfusion,” Journal of Magnetic Resonance Imaging 60, no. 1 (2024): 245–255, 10.1002/jmri.29094. 37881827

[12] D. L. Longo , A. Busato , S. Lanzardo , F. Antico , and S. Aime , “Imaging the pH Evolution of an Acute Kidney Injury Model by Means of Iopamidol, a MRI‐CEST pH‐Responsive Contrast Agent,” Magnetic Resonance in Medicine 70, no. 3 (2013): 859–864, 10.1002/mrm.24513. 23059893

[13] Q. Zhang , Q. Tao , Y. Xie , et al., “Assessment of Rhabdomyolysis‐Induced Acute Kidney Injury With Chemical Exchange Saturation Transfer Magnetic Resonance Imaging,” Quantitative Imaging in Medicine and Surgery 13, no. 12 (2023): 8336–8349, 10.21037/qims-23-699. 38106319 PMC10722020

[14] D. L. Longo , J. C. Cutrin , F. Michelotti , P. Irrera , and S. Aime , “Noninvasive Evaluation of Renal pH Homeostasis After Ischemia Reperfusion Injury by CEST‐MRI,” NMR in Biomedicine 30, no. 7 (2017): 1–8, 10.1002/nbm.3720. 28370530

[15] K. Pavuluri , I. Manoli , A. Pass , et al., “Noninvasive Monitoring of Chronic Kidney Disease Using pH and Perfusion Imaging,” Science Advances 5, no. 8 (2019): eaaw8357, 10.1126/sciadv.aaw8357. 31453331 PMC6693904

[16] D. Kentrup , P. Bovenkamp , A. Busch , et al., “GlucoCEST Magnetic Resonance Imaging In Vivo May Be Diagnostic of Acute Renal Allograft Rejection,” Kidney International 92, no. 3 (2017): 757–764, 10.1016/j.kint.2017.04.015. 28709641

[17] Y. Wu , I. Y. Zhou , T. Igarashi , D. L. Longo , S. Aime , and P. Z. Sun , “A Generalized Ratiometric Chemical Exchange Saturation Transfer (CEST) MRI Approach for Mapping Renal pH Using Iopamidol,” Magnetic Resonance in Medicine 79, no. 3 (2018): 1553–1558, 10.1002/mrm.26817. 28686805 PMC5756701

[18] H. Kim , Y. Wu , D. Villano , D. L. Longo , M. T. McMahon , and P. Z. Sun , “Analysis Protocol for the Quantification of Renal pH Using Chemical Exchange Saturation Transfer (CEST) MRI,” Methods in Molecular Biology 221 (2021): 667–688, 10.1007/978-1-0716-0978-1_40. PMC9703203 33476030

[19] K. D. Pavuluri , L. Consolino , D. L. Longo , P. Irrera , P. Z. Sun , and M. T. McMahon , “Renal pH Mapping Using Chemical Exchange Saturation Transfer (CEST) MRI: Experimental Protocol,” Methods in Molecular Biology 221 (2021): 455–471, 10.1007/978-1-0716-0978-1_27. PMC9703269 33476017

[20] D. L. Longo , P. Irrera , L. Consolino , P. Z. Sun , and M. T. McMahon , “Renal pH Imaging Using Chemical Exchange Saturation Transfer (CEST) MRI: Basic Concept,” Methods in Molecular Biology 221 (2021): 241–256, 10.1007/978-1-0716-0978-1_14. PMC9703214 33476004

[21] J. Keupp , E. Heijman , S. Langereis , et al., “Respiratory Triggered Chemical Exchange Saturation Transfer MRI for pH Mapping in the Kidneys at 3T,” in Proceedings of the 19th Annual Scientific Meeting ∖& Exhibition of the International Society for Magnetic Resonance in Medicine (ISMRM Web Editorial Board, 2011).

[22] P. Indic , D. Paydarfar , and R. Barbieri , “A Point Process Model of Respiratory Dynamics in Early Physiological Development,” in 2011 Annual International Conference of the IEEE Engineering in Medicine and Biology Society (IEEE, 2011), 3804–3807, 10.1109/IEMBS.2011.6090771. PMC3340562 22255168

[23] H. Groeben , S. Meier , C. G. Tankersley , W. Mitzner , and R. H. Brown , “Heritable and Pharmacological Influences on Pauses and Apneas in Inbred Mice During Anesthesia and Emergence,” Experimental Lung Research 31, no. 9–10 (2005): 839–853, 10.1080/01902140600586458. 16684716

[24] O. F. C. van den Bosch , R. Alvarez‐Jimenez , H. J. de Grooth , A. R. J. Girbes , and S. A. Loer , “Breathing Variability—Implications for Anaesthesiology and Intensive Care,” Critical Care 25, no. 1 (2021): 280, 10.1186/s13054-021-03716-0. 34353348 PMC8339683

[25] J. W. Carlson and D. M. Kramer , “Rapid Radiofrequency Calibration in MRI,” Magnetic Resonance in Medicine 15, no. 3 (1990): 438–445, 10.1002/mrm.1910150309. 2233222

[26] W. H. Perman , M. A. Bernstein , and J. C. Sandstrom , “A Method for Correctly Setting the Rf Flip Angle,” Magnetic Resonance in Medicine 9, no. 1 (1989): 16–24, 10.1002/mrm.1910090104. 2709993

[27] P. A. Yushkevich , J. Piven , H. C. Hazlett , et al., “User‐Guided 3D Active Contour Segmentation of Anatomical Structures: Significantly Improved Efficiency and Reliability,” NeuroImage 31, no. 3 (2006): 1116–1128, 10.1016/j.neuroimage.2006.01.015. 16545965

[28] I. Y. Zhou , E. Wang , J. S. Cheung , X. Zhang , G. Fulci , and P. Z. Sun , “Quantitative Chemical Exchange Saturation Transfer (CEST) MRI of Glioma Using Image Downsampling Expedited Adaptive Least‐Squares (IDEAL) Fitting,” Scientific Reports 7, no. 1 (2017): 84, 10.1038/s41598-017-00167-y. 28273886 PMC5427899

[29] J. Windschuh , M. Zaiss , J. Meissner , et al., “Correction of B 1‐Inhomogeneities for Relaxation‐Compensated CEST Imaging at 7 T,” NMR in Biomedicine 28, no. 5 (2015): 529–537, 10.1002/nbm.3283. 25788155

[30] G. L. Simegn , P. Z. Sun , J. Zhou , et al., “Motion and Magnetic Field Inhomogeneity Correction Techniques for Chemical Exchange Saturation Transfer (CEST) MRI: A Contemporary Review,” NMR in Biomedicine 38, no. 1 (2025): e5294, 10.1002/nbm.5294. 39532518 PMC11606773

[31] P. Virtanen , R. Gommers , T. E. Oliphant , et al., “SciPy v1.11.1: Fundamental Algorithms for Scientific Computing in Python,” Nature Methods 17, no. 3 (2020): 261–272, 10.1038/s41592-019-0686-2. 32015543 PMC7056644

[32] J. Stabinska , A. Müller‐Lutz , H. J. Wittsack , et al., “Two Point Dixon‐Based Chemical Exchange Saturation Transfer (CEST) MRI in Renal Transplant Patients on 3 T,” Magnetic Resonance Imaging 90 (2022): 61–69, 10.1016/j.mri.2022.04.004. 35476934

[33] J. Lu , J. Zhou , C. Cai , S. Cai , and Z. Chen , “Observation of True and Pseudo NOE Signals Using CEST‐MRI and CEST‐MRS Sequences With and Without Lipid Suppression,” Magnetic Resonance in Medicine 73, no. 4 (2015): 1615–1622, 10.1002/mrm.25277. 24803172 PMC4223020

[34] T. K. Koo and M. Y. Li , “A Guideline of Selecting and Reporting Intraclass Correlation Coefficients for Reliability Research,” Journal of Chiropractic Medicine 15, no. 2 (2016): 155–163, 10.1016/j.jcm.2016.02.012. 27330520 PMC4913118

[35] F. Charlier , M. Weber , S. Proost , et al., Statannotations (Zenodo, 2024), 10.5281/zenodo.14258156.

[36] J. Stabinska , P. Neudecker , A. Ljimani , H. Wittsack , R. S. Lanzman , and A. Müller‐Lutz , “Proton Exchange in Aqueous Urea Solutions Measured by Water‐Exchange (WEX) NMR Spectroscopy and Chemical Exchange Saturation Transfer (CEST) Imaging In Vitro,” Magnetic Resonance in Medicine 82, no. 3 (2019): 935–947, 10.1002/mrm.27778. 31004385

[37] A. Liebeskind , J. R. Schüre , M. S. Fabian , et al., “The Pulseq‐CEST Library: Definition of Preparations and Simulations, Example Data, and Example Evaluations,” Magnetic Resonance Materials in Physics, Biology and Medicine 38, no. 3 (2025): 413–422, 10.1007/s10334-025-01242-6. PMC12255581 40146474

[38] E. Vinogradov , L. Zheng , M. Ananth , et al., Endogenous Urea CEST (urcest) for MRI Monitoring of Kidney Function.

[39] F. Schmitz‐Peiffer , M. Lukas , A. M. Mohan , et al., “Effects of Isoflurane Anaesthesia Depth and Duration on Renal Function Measured With [99mTc]Tc‐Mercaptoacetyltriglycine SPECT in Mice,” EJNMMI Research 14, no. 1 (2024): 4, 10.1186/s13550-023-01065-3. 38180547 PMC10769950

[40] H. M. Costello , J. G. Johnston , A. Juffre , G. R. Crislip , and M. L. Gumz , “Circadian Clocks of the Kidney: Function, Mechanism, and Regulation,” Physiological Reviews 102, no. 4 (2022): 1669–1701, 10.1152/physrev.00045.2021. 35575250 PMC9273266

[41] H. Qi , C. Ø. Mariager , J. Lindhardt , P. M. Nielsen , H. Stødkilde‐Jørgensen , and C. Laustsen , “Effects of Anesthesia on Renal Function and Metabolism in Rats Assessed by Hyperpolarized MRI,” Magnetic Resonance in Medicine 80, no. 5 (2018): 2073–2080, 10.1002/mrm.27165. 29520870

[42] A. Mercatello , “Modifications de la fonction rénale induites par l'anesthésie,” Annales Françaises d'Anesthèsie et de Rèanimation 9, no. 6 (1990): 507–524, 10.1016/S0750-7658(05)80223-0. 2278418

[43] B. Ø. Christoffersen , C. J. Bundgaard , K. R. Hjøllund , et al., “Influence of General Anaesthesia on Circulating Biomarkers of Glucose Metabolism in Pigs,” Laboratory Animals 57, no. 6 (2023): 650–663, 10.1177/00236772231187179. 37647768

[44] R. Frithiof , O. Soehnkein , S. Eriksson , et al., “The Effects of Isoflurane Anesthesia and Mechanical Ventilation on Renal Function During Endotoxemia,” Acta Anaesthesiologica Scandinavica 55, no. 4 (2011): 401–410, 10.1111/j.1399-6576.2011.02406.x. 21391922

[45] K. L. Kong , J. E. Tyler , S. M. Willatts , and C. Prys‐Roberts , “Isoflurane Sedation for Patients Undegoing Mechanical Ventilation: Metabolism to Inorganic Fluride and Renal Effects,” British Journal of Anaesthesia 64, no. 2 (1990): 159–162, 10.1093/bja/64.2.159. 2317417

[46] A. Müller‐Lutz , A. Ljimani , J. Stabinska , et al., “Comparison of B0 Versus B0 and B1 Field Inhomogeneity Correction for Glycosaminoglycan Chemical Exchange Saturation Transfer Imaging,” Magnetic Resonance Materials in Physics, Biology and Medicine 31, no. 5 (2018): 645–651, 10.1007/s10334-018-0689-5. 29761413

[47] C. Stilianu , C. Graf , M. Huemer , et al., “Enhanced and Robust Contrast in CEST MRI: Saturation Pulse Shape Design via Optimal Control,” Magnetic Resonance in Medicine 92, no. 5 (2024): 1867–1880, 10.1002/mrm.30164. 38818538

[48] M. Wagner , C. Klessen , M. Rief , et al., “High‐Resolution t2‐Weighted Abdominal Magnetic Resonance Imaging Using Respiratory Triggering: Impact of Butylscopolamine on Image Quality,” Acta Radiologica 49, no. 4 (2008): 376–382, 10.1080/02841850801894806. 18415778

[49] B. Li , H. She , S. Zhang , et al., “Image Registration With Structuralized Mutual Information: Application to CEST,” in Proceedings of the 25th Annual Meeting of the International Society for Magnetic Resonance in Medicine (ISMRM) (ISMRM Web Editorial Board, 2017), 1293, https://cds.ismrm.org/protected/17MProceedings/PDFfiles/1293.html.

[50] M. Ragnarsdóttir and E. K. Kristinsdóttir , “Breathing Movements and Breathing Patterns Among Healthy Men and Women 20–69 Years of Age,” Respiration 73, no. 1 (2006): 48–54, 10.1159/000087456. 16106113

[51] K. J. Layton , S. Kroboth , F. Jia , et al., “Pulseq: A Rapid and Hardware‐Independent Pulse Sequence Prototyping Framework,” Magnetic Resonance in Medicine 77, no. 4 (2017): 1544–1552, 10.1002/mrm.26235. 27271292

